# Intensive care nurses’ experiences of teamwork during the covid-19 pandemic. a qualitative study.

**DOI:** 10.1186/s12912-025-02696-8

**Published:** 2025-01-27

**Authors:** Jeanette Eckerblad, Åsa Dorell, Helen Conte

**Affiliations:** Department of Neurobiology, Care Sciences and Society, Karolinska Institutet, Nobels Allé 23 Huddinge, Stockholm, SE-141 83 Sweden

**Keywords:** Intensive care units, Nursing, Team, Transition, Pandemics, Qualitative research

## Abstract

**Background:**

Teamwork is a core competence for all health care professionals and quality of care is a vital outcome. The pandemic backdrop of 2020–2022 led to initial chaos and adaptation of the nurses’ roles and responsibilities in the intensive care unit. Therefore, the purpose was to describe the intensive care nurses’ experiences of working in teams during the Covid-19 pandemic and discussing the results through the lens of transitiontheory.

**Methods:**

Individual and semi-structured interviews were conducted with 16 intensive care nurses. The interview transcripts were analysed using Braun & Clarke’s six-step inductive thematic analysis.

**Results:**

The intensive and critical care nurses’ experiences during the first 18 months of the Covid-19 pandemic captured chronological and conceptual commonalities, which were represented in three themes, “*Losing the security of the ICU team”*,* “Having time to adapt and finding structure for collaborative work”*,* and* “*Gaining professional growth through adapting collaborative work to contextual challenges*”.

**Conclusion:**

Losing the security of the intensive care unit team, having to adapt to constant changes, and the need to provide care to an increased number of critically ill patients led to a sense of being left to manage on their own. The intensive care unit nurses missed the interprofessional collaboration that had previously been a core part of their professional role. The adaptability and willingness to find solutions helped nurses regain control, manage the challenges they faced and find new ways to collaborate.

**Clinical trial number:**

Not applicable.

**Supplementary Information:**

The online version contains supplementary material available at 10.1186/s12912-025-02696-8.

## Background

Teamwork is a core competence for all health care professionals and quality of care is a vital outcome [1]. The intensive care team is fluid, it expands and contracts from a small core bedside team to a larger extended team based on the patients’ care needs [2, 3]. Caring together for critically ill patients with organ failure in the intensive care unit (ICU) can be high stake and team interaction in activities ranges from collaboration to conflict [2]. Interprofessional collaboration (IPC) is a process where members from diverse professions with different levels of experience contribute to a combined expertise by working and reflecting together [1]. Being able to maintain control together in their interplay is vital to experiencing well-functioning collaboration [4, 5]. IPC is a mechanism for teamwork and when members understand each other’s roles, they plan and coordinate their work together [6]. One member’s expertise is acknowledged by others through the negotiation of independent and interdependent goals into patient centred care [7, 8]. Role clarity in the collaborative interplay contributes to the reinforcement of each other’s goals in joint planning and problem solving [1, 6]. The micro and macro environments and organizational values affect IPC and can serve both as facilitators and barriers for the quality of IPC [1].

The pandemic backdrop of 2020–2022 led to initial chaos and adaptation of the nurses’ roles and responsibilities in the ICU [9–11]. Research has suggested that the large influx of critically ill patients necessitated extending the ICUs’ capacity, and the nurses’ role changed [9–14]. Intensive care nurses suggested that both the quality and nature of care changed during the initial wave of the Covid-19 pandemic [9–14]. Nurses also suggested that standards of care were affected, and care became fragmented and less holistic [9, 10, 14]. Nurses suggested suboptimal care had an impact on the patients’ progression and recovery [14].

The initial stages of Covid-19 created new work structures (9). The lack of ICU nurses led to reorganization of hospital staff and introduced skills mix, where the ICU nurses worked in small beside teams as well as taking on the supervising and consulting of less experienced nurses or nurses without ICU specialization [11, 14]. The ICU nurses’ increased workload led to them having to let go of important roles and assignments, for example providing and coordinating information and support for patients’ family members, and technological and team-based solutions were implemented [15–18]. New assignment-based groups were working alongside or separate from the ICU team, for example, prone position teams [14]. These changes can be viewed as a transition for the ICU nurses. The concept involves moving from one life situation to another, characterized as a process initiated by a change in an individual’s life that necessitates time and understanding from both the individual and those in their environment [19].

Transition Theory is based on individuals’ experiences of transitions that occur through altered situations and conditions [20]. Transition describes a person’s responses to a change during the time the change is happening [21] and involves both a process that engages the person’s psychological ability to adapt to a changed circumstances and the outcome of the change. Transitions can be voluntary or forced by external circumstances. A transition begins when a person becomes conscious of a life altering event, defined as a critical point [21], and the individual undergoes a transitional process and incorporates the altered experiences into their life [22].

The experiences gained from this period ought to be described and documented so that healthcare providers and their organizations could learn from this while preparing for future pandemics or crises. Therefore, the aim of this study was to describe the ICU nurses’ experiences of working in teams during the Covid-19 pandemic.

## Method

A descriptive qualitative study using an inductive approach. Part of the data from the qualitative interviews have previously been published in an article focusing on nurses’ experiences of communicating with and supporting relatives from a distance while working during visiting restrictions [18]. All parts of the study followed the ethical guidelines and have been approved by the national Swedish Ethical Review Authority (DNR 2020–05961). The study was reported in adherence to the consolidated criteria for reporting qualitative research guidelines (COREQ) [23].

### Participants and settings

Intensive care nurses from four different intensive care departments in Sweden were enrolled in the study through a convenience sample in 2021. The ICU departments were located in different parts of Sweden, two in the north and two in the south. The hospital included two university hospitals and two inner-city hospitals and differed in size and level of care.

Written information and permission to conduct the study was sent to the head of the departments. To identify prospective participants, the heads of nursing informed the staff of the prospective study. ICU nurses who were interested and met the inclusion criteria, i.e., postgraduate education in intensive care and worked in the ICU during Covid-19 pandemic, received written information regarding the study. Participants signed written consent and a total of 19 ICU-nurses were interested in participating and were contacted by email. Sixteen ICU-nurses agreed to be interviewed, 12 women and four men. They had worked in the ICU department for a median of nine years, with a range from 1 to 28 years of work experience (See table 1).

**Table 1:** Characteristics of the participants

Code number	Sex	Postgraduate education, intensive care	Years as ICU specialist nurse	Type of hospital
1	Female	x	21	Small inner-city hospital
2	Male	x	22	Small inner-city hospital
3	Male	x	14	Small inner-city hospital
4	Female	x	23	Large inner-city hospital,
5	Feamle	x	28	Large inner-city hospital,
6	Female	x	9	Large inner-city hospital,
7	Female	x	5	Large inner-city hospital,
8	Male	x	1	University hospital
9	Female	x	3	University hospital
10	Female	x	17	University hospital
11	Male	x	1	University hospital
12	Female	x	2	University hospital
13	Female	x	9	University hospital
14	Female	x	8	University hospital
15	Female	x	11	University hospital
16	Female	x	9	University hospital

### Data collection

A semi-structured interview guide (see appendix 1) was developed with the purpose to explore the ICU nurses’ experiences of working in ICU during the pandemic. The interviews were conducted through video conferences during 2021 and the first half of 2022 by the authorsof the study. A first test interview was conducted initially, and no changes were made to the interview guide afterthe authors reviewed the structure, style and outcome. The interviews lasted between 20 and 56 min, with a median of 41 min. The interviews were digitally recorded with permission from the participants, and transcribed verbatim. The initial questions focused on demographic data of the participants. Questions from the guide which were later analysed in the current study included: How did intensive care change during the Covid-19 pandemic? How did the collaboration in your workgroup change during the Covid-19 pandemic? How did your situation as an intensive care nurse change during the Covid-19 pandemic? The authors used follow-up questions, such as “How do you think it worked?”, “Do you want to describe it?” “How did that feel for you”?

### Data analysis

The interview transcripts were analysed using Braun & Clarke’s [24, 25] six-step inductive thematic analysis. To become familiar with the data, the transcribed interview transcripts were read and re-read by three authors in the first step of the analysis process (JE, ÅD and HC) several times. All authors took notes and marked initial ideas and started generating codes. In the second step, coded extracts from the entire dataset were reviewed, and codes were independently generated and refined by the first author (JE). Passages of data relevant to the codes were collected in a separate document. The codes were reviewed and sorted into to three domains of team, goal, and roles by the last author (HC), and a concept map was constructed. In the third step, the codes were grouped into potential subthemes and themes by two of the authors (JE, HC) by moving from the whole dataset and collapsing the material. In the fourth step, the same two authors refined the themes and subthemes together and produced the final thematic map. Both authors went back to the original transcripts and read them once more to confirm the structured analysis framework. In the fifth step, two authors (JE, HC) took turn in reading and refining the sub themes and then reviewed and condensed the themes to three, with six sub-themes (See Table 2). One author (ÅD) initially checked the consistency and relationship between emerging themes and subthemes in steps three, four and five. The final structure was created after all authors (JE, ÅD, HC) had checked and reviewed the data. The sixth and final step was to produce the report (JE, ÅD, HC). All co-authors read and commented on the results.

**Table 2:** An overview of the findings

Themes	Subthemes
Losing the security of the ICU team	To be left to manage on my own
	To keep up when everything changes
Having time to adapt and finding structure for collaborative work	To gain a new organizational structure
	To find new ways for collaboration
Gaining professional growth through adapting collaborative work to contextual challenges	To regain quality of collaborative care
	To offer each other emotional support

#### Findings

The nurses’ experiences from working in the ICU´s during the first 18 months of the Covid-19 pandemic consisted of commonalities and variations. The interviews captured chronological and conceptual commonalities, which were represented in three themes of “*Losing the security of the ICU team”*,* “Having time to adapt and finding structure for collaborative work”*,* and “Gaining professional growth through adapting to contextual challenges”* (See table 2).

The interviews suggested that the ICU nurses transitioned from experiencing losses to be able to manage the influx of patients, to experiencing gain through what it was possible to achieve at a later stage while working together with other professions in the ICU. Regional variations framed the themes, e.g., number of patients admitted, when each wave started, time in and between each wave, as well individual variations represented by the amount of pressure put on the nurses’ work in the ICU team.

### Losing the security of the ICU team

This theme describes how nurses lost both their own role in the team and the security of the interprofessional competence that the core ICU team represented for them. The nurses’ experiences revealed what impact the loss of a secure ICU team had when caring for an increased number of critically ill patients. The theme includes two sub-themes: ‟*To be left to manage on my own”* and *“To keep up when everything changes”.*

#### To be left to manage on my own

The large influx of critically ill patients to the ICUs in immediate need of both respiratory and circulatory support affected team structures. The ICU teams, where each professional had well-defined role, and was present in bedside care and in coordinative activities, were broken up. Some professions went from being present and active to being absent or with limited physical presence in units within days of the pandemic breaking out.

*Quote. “Yes*,* it was difficult. My ICU team*,* it disappeared*,* completely.”* (Participant 15).

Nurses also lost the secure ICU setting and had to work in temporary wards outside the ICU, or in adapted rooms set up within ICU to accommodate the care of a larger number of patients in isolation, and with a reduced number of staff. The participants narrated that they had lost what was previously known to them, which was working in close proximity to, and together with, other ICU specialists in interprofessional collaborative care.

*Quote.* ‟*In the beginning*,* we didn’t collaborate at all. We didn’t have time*,* everyone was working on their own just trying to put out fires*,* so to speak.”* (Participant 9).

There was a large influx of new practitioners, nurses, auxiliary nurses and physicians, both with and without specialist training in other fields, who were relocated to ICU. The formal and informal competence of each new person was initially unknown. The new staff, with limited training and introduction, were put into managing situations with critically ill ICU patients. The ICU nurses experienced being split between handling an increased amount of critically ill patients and trying to supervise other practitioners from different professions with limited intensive care experience, both in bedside and coordinative care.

*Quote. “I worked with one assistant nurse who had previous ICU experience*,* three assistant nurses from the anaesthesiology department*,* who were quite newly introduced to our ICU*,* and three more assistant nurses from anaesthesiology department who had never worked with ICU patients before. So*,* on the one hand*,* I should manage three critically ill covid patients*,* and on the other hand supervise staff with no previous ICU experience.”* (Participant 16).

#### To keep up when everything changes

The ICU nurses experienced the first wave as chaotic. The large influx of critically ill patients with an initially unknown disease led to feelings of uncertainty in care, new management and treatments which were continuously introduced to limit patients’ suffering. The availability of medications such as sedatives were limited, and standards of care changed continuously. New guidelines were set up, and not knowing standards of care was difficult for the nurses to handle. The ICU staff were busy trying to save lives and minimize patients’ suffering. They were unable to easily foresee and plan for possible care trajectories. The participating ICU nurses had to shift focus from what they believed was right and important, to what was possible right there and then.

*Quote. “The conditions and directives on how we could and should work could change the same day*,* a certain thing that we had planned before lunch could already have been changed in the afternoon. This caused irritation*,* ‘What is the matter now? Should we do this or should we that?’ I had to be flexible and change all the time.” (Participant 3)*.

To work preventively, for example to avoid pressure ulcers, had to be low priority in order to deal with more urgent problems and life-threatening outcomes. Ensuring the patients’ survival using the least possible resources became the primary focus. Nurses felt conflicted, since they experienced that the quality of care deteriorated due to the lack of time and opportunity to coordinate and plan their care collaboratively with the rest of the ICU team. They also felt conflicted because they understood the shift away from prevention and collaborative care would cause both short- and long-term suffering for the patients and their families. The ICU nurses’ work kept them in close proximity to patients and it was stressful to observe and continuously identify risks in patients’ care and in collaborative care activities. Many of the participants were afraid or unable to leave the patients. They felt that what they did was not enough, either for the patients or supporting the new professionals working in ICU. The ICU nurses realized that they had to shoulder a heavy burden and responsibility to make things work.

*Quote. “In the beginning*,* I had to control everything with a firm hand*,* because I did not know who I had in front of me*,* physician*,* nurses*,* or assistant nurses. If I needed help with medications*,* for example*,* I always asked ‘Do you know how to do this? Do you have experience of how to prepare this?’ Because I didn’t know who I had in front of me. It wasn’t a good collaboration really*,* so*,* it was difficult.”* (Participant 9).

The ICU nurses initially experienced that their role changed due to the challenges of caring for an increased number of critically ill patients and working together with less experienced staff. They lost parts of their own core role, which consisted of supporting and coordinating support for the family members. They had to watch and accept other professions taking over what was important to them. They were overwhelmed but realized they had to prioritize managing and leading the bedside care. Their role in bedside care extended, and they felt as though they were responsible for the quality of care of their own patients, and for other groups of patients. They focused on using their knowledge to support and coordinate the work of other nurses in ICU.

*Quote.* ‟*Everyone kind of knew that we had to roll up our sleeves and help each other out*,* in order to get through this*,* and cross-professionally as well. A lot of people had to do many things that they might not*,* that they never had done before. But everyone helped each other*,* supported*,* and guided each other as much as they possibly could.”* (Participant 14).

### Having time to adapt and finding structure for collaborative work

This theme describes how the participants tried to adapt, develop, and organize the way they worked and collaborated in between the peaks of the different waves. The theme includes two sub-themes: *“To gain a new organizational structure”*, and *“To find new ways for collaboration”.*

#### To gain a new organizational structure

Participants described the 18-month period as a time where they tried to adapt their work to meet challenges during the peaks of the different waves of the pandemic. They tried to regain structure and establish ways to organize their work. The high influx of patients with Covid-19 had led to the ICU nurses starting to gain knowledge and experience of how to care for and manage the problems of these severely ill patients.

*Quote. “Intensive care changed in so many ways*,* patients required heavy intensive care and more of everything*,* higher settings of the ventilator*,* more sedative drugs*,* and it required more staff to manage.”* (Participant 13).

The fear of being infected or infecting others had declined. The ICU nurses started to look at the future in a new and more positive way. They had moved on from the initial uncertainty and the feeling of being forced into a role against their own will. With more knowledge and experience, the nurses started to gain routines and strategies when caring for patients suffering from Covid-19. The organization had adapted and prepared new treatments and care to fit the needs of these patients, and had gained knowledge and experience from how the patients reacted and responded to treatments. They had started to find ways to structure and organize their work in order to handle the influx and manage the care of the many patients.

*Quote.* ‟*Then it became more and more structured during the second and third waves. Things started to fall in to place*,* we began to figure out how to treat these patients*,* and we had more premises prepared for ICU care*,* so we could take on a larger volume of patients.”* (Participant 16).

During the first wave of the pandemic, the participants described a lack of teamwork, and that the collaborative interplay between the different professions was practically non-existent. Gradually, this changed and new teams were formed, their work went from being chaotic to manageable. Participants described that although they had faced a chaotic situation, these challenges had led to several creative solutions among the co-workers and a problem-solving environment in order to regain structure.

*Quote. “After all*,* there have been huge developments regarding the creativity of people to help find solutions.”* (Participant 2).

#### To find new ways for collaboration

With time to reflect and adapt, the participants described that they and their organizations started to find new structures of collaboration with other wards to support the ICUs with healthcare personnel.

*Quote. “We got help from other wards*,* nurses both with and without specialist education. They needed a lot of supervision*,* but they followed our routines and did what they were supposed to.”* (Participant 13).

After the initial influx of patients, the staff who were rotated into the Covid ICU did so on voluntary basis. The ICU nurses expressed they were grateful for the extra pairs of hands, minds and hearts of those new co-workers who chose to support the ICU and patient care with their competence. The participants described that the teamwork and collaboration worked better and that they apricated the motivation and the courage of the staff, and that this was a vital part in forming new teams.

*Quote. “It’s not that easy to just join in and start working*,* they got like*,* a two-day introduction and then they were just supposed to. No*,* but the ones who came during the second wave*,* they were very motivated. After all*,* they had been given a choice*,* and they chose to come to the ICU. And after that*,* everything went smoothly. They became that part of the team very quickly*,* and it felt good for all of us.”* (Participant 6).

Professionals with ICU competence was divided to cover all new teams that had to be created when the number of patients drastically increased again in the ICU. To constantly work with new colleagues whom, they did not know had initially been stressful. But, with time, the ICU nurses gradually gained an understanding of the competence of the new staff and how the responsibility and assignments could be divided between them to increase quality and safety of patient care. The ICU’s specialist nurses, and auxiliary nurses were matched with other professionals without formal, or limited, ICU competence in bedside care and coordination experience, to ensure quality of care. They supported and supervised other professions in bedside care.

*Quote. “And then we had decided that … a team would not consist of e.g.*,* an anaesthesiology nurse and an assistant nurse from the operation room department*,* but that we should always divide the competence so that e.g.*,* ICU nurses worked with an OR assistant nurse and the ICU assistant nurse worked with an anaesthesiology nurse.”* (Participant 4).

### Gaining professional growth through adapting collaborative work to contextual challenges

The theme describes how the participants had learned from working under the circumstances that the pandemic presented; they reflected on the way they had navigated through new challenges, expanded their skills and responsibilities. The theme includes two sub-themes, *“To regain quality of collaborative care”*, and “*To offer each other emotional support”.*

#### To regain quality of collaborative care

When things started to settle, participants with extensive experience of working in the ICU teams before the pandemic wanted to bring back the collaborative and patient-centred care they once had, but which they felt had been lost during the pandemic. They expressed that they wanted to get back to planning proactive and goal-oriented care collaboratively, and to working as interprofessional teams.

*Quote. “I’ve missed what had been before everything was set on pause. To collaborate and plan forward*,* not just extinguishing fires.”* (Participant 9).

During the pandemic, the ICU had been struggling with a high workload and shortage of co-workers. The participants described that they learned to grab the moment as it appeared, they learned how to be flexible and to find creative solutions to be able to meet challenges. Before the pandemic they had been routine oriented, doing things in a certain way and at a certain time, e.g., help the patient with hygiene and making the bed in the morning, or mobilizing them to a seated position in the afternoon. Now, when all routines were set aside, they had learned to focus on the now, and to question routines; if they had the manpower to do it, they did it, since they there were no benefits to waiting.

*Quote. “So these things with mobilization*,* we have got a completely different approach to that now; if there are enough people around in the morning before anything else has happened*,* or even before we have started our shift*,* we will do it.”* (Participant 2).

The participants reflected on the importance of the interprofessional ICU teams, how they had worked together before the outbreak, when everyone knew what was expected of them and they knew what to do. This was in contrast to how it had worked during the pandemic; the participants concluded that new colleagues needed a structured introduction and time to learn how to manage, plan and lead collaborative care. The healthcare professionals who started during the pandemic with no previous ICU experience initially only met Covid-19 patients and only knew Covid care, which was not in line with the quality care the ICU nurses had and which they now wanted to offer their patients. Some of the new co-workers were offered a second introduction when things started to settle down. They were introduced to care bundles and protocols that had previously been used in ICU before the pandemic outbreak.

*Quote. “And these new co-workers*,* they have never been in contact with*,* or ever met*,* real ICU care before. And I don’t want to say that pandemic care was real ICU care. There are many of us … especially among the ICU nurses*,* who are concerned about that part; about what will happen.*” (Participant 15).

#### To offer each other emotional support

The participants described they had learned the importance of a secure and friendly work environment. They needed each other, and as a group they had become more sensitive and supportive of one another. What they had been through and experienced during the pandemic was horrible in every sense. They were fully aware that the pandemic had taken a toll on every one of them in different ways, and people had been affected on levels that should never have happened in a workplace.

*Quote. “Together we have been through something that can be compared to a disaster. And that strengthens a group*,* hopefully.”* (Participant 13).

They now talked more to each other about other things than they did before the pandemic started. Everyone had their own story and experiences of the crises or disaster that they had gone through. They tried to be there for each other, to confirm, guide and advise.

*Quote. “I think we have become kinder and more supportive of each other*,* trying to reduce the risk of illness among colleagues.”* (Participant 9).

The participants had to navigate and manage several challenges through the pandemic; their old teams were split up, and they have all had a lot of new colleagues during these last 18–20 months. Forming new teams had made the participants realize how important their old team and team members were to them. Not only for the quality of the collaboration they had while working together in bedside care, but also for the personal and emotional support the teams offered.

*Quote. “It feels like the cooperation is better now among our old team members. We are tighter*,* more tolerant*,* and very kind to each other.”* (Participant 13).

To sum it up, when the participants finally dared to look forward and started to make long-term plans, they realized that looking ahead also required them to look back on what they had been through, and how they needed to prepare if something similar were to occur in the future.

*Quote.“I think I have probably landed now somehow*,* in retrospect*,* thinking of all that has happened I’m absolutely sure that we can never*,* never let it happen again*,* and if it does*,* we need another approach.”* (Participant 15).

## Discussion

This study explored ICU nurses’ experiences of working during the first 18 months of the Covid-19 pandemic. The findings in the current study show that it was a life-changing event, and a process that initially entailed managing a large influx of critically ill patients while simultaneously losing the security of the core ICU team and the secure ICU environment. The findings in the current study suggested the extraordinary circumstances caused emotional ambivalence and stress, which has been confirmed by ICU nurses in other studies [9, 13, 14, 26]. During the initial 18 months of the pandemic, ICU nurses in the current study suggested that they moved from experiencing chaos, to facing something unknown, and integrating what they had learned to help them face future challenges. Transition theory can be applied to understand how the ICU nurses navigated through the extraordinary circumstances caused by the pandemic. A transition occurred when they started to gain experience from what was necessary in chaos, to understanding what was possible while working in groups and teams in the ICU. This is in line with Kralik, Visentin & Van Loon [22], who suggest that situational transitions lead to changes in nurses’ role due to changing events.

A transition can be divided into three phases: separation, transition, and integration [22]. The separation phase entails a breaking point where individuals are in crisis due to a change in a situation. This phase can be frustrating, and individuals can experience uncertainty in their altered role [19, 22]. The initial shock during the Covid-19 pandemic was represented by the sudden influx of critically ill patients in need of ventilatory and circulatory support, but also a breakdown of well-established team structures which led to a forced change for the nurses in the current study. These findings are supported by a previous study by Ambrose et al. [27]. Our findings are also in line with the concept of separation, where the known and familiar were disrupted. The nurses experienced the loss of the secure ICU team, where the different professions had well-defined roles in bedside care in the safe ICU setting, and the nurses was left supervising different professions with limited ICU experiences. They felt that they were not doing enough and understood that the choices they had to make to save as many lives as possible in the prevailing situation would cause patients long-term suffering. These findings are in line with previous research, which showed that roles and responsibilities expanded to encompass caring of several patients at the same time, coordinating care and concurrently introducing and supervising temporary colleagues [26].

Separation from known roles and responsibilities is a breaking point where the individuals in crisis respond to a change in situation or condition, which is frustrating and can lead to feelings of uncertainty and confusion [19, 22]. Nurses in other studies have suggested that role extension or to change core responsibilities was necessary but stressful due to the extraordinary circumstances [9–11, 18, 26]. Separation also led to the loss of ICU nurses’ coordinative role in person-centred care. The nurses in the current study expressed both ambivalence and frustration since they could not do what needed to be done for the critically ill patients or their families who were separated from each other. The nurses also expressed uncertainty in care and treatment since it was continuously adapted to limit patient suffering with the resources available. This is in line with studies in which healthcare professionals described unfamiliarity of the infectious ailment, limited experience, and a lack of scientific evidence concerning treatment and prognosis [26], This shift caused ambivalence; the participants did not have enough experience to predict how the individual patient might respond, and there were not enough resources to individualize care. These factors were experienced as an intensifying pressure, stress and hindering the ability to handle care. However, the influx of patients with the same diagnosis also quickly facilitated standardization of care [26]. The situation forced the ICU nurses to weigh the need of the individual patient against what was necessary for the group of patients in ICU. The shift entailed focusing on saving lives and reducing short-term suffering for as many patients as possible, using the least amount of resources. Nurses suggested it was like being on an assembly line, with limited possibility to provide proactive nursing care [9], and where they had to use their entire intellectual capacity to adapt [13].

Nurses in the current study initially experienced that they lost all that was known to them and that they had moved into the unknown. They struggled to adapt to evolving practice and new guidelines. Other studies suggested that ICU nurses felt forced to leave behind the good standard of care they had been used to providing [10, 11] and that communication in the team was a challenge, as well as communication between teams during handovers [14]. The shift from proactive to reactive approach, primarily focused on immediate patient survival, reflects the adaptive response during the first chaotic phase [27]. Separation is followed by the transition phase, where people find themselves between two phases [22]. This phase can be described as a no man’s land, where the person faces a transition to something unknown. It involves an emotional phase, where the individual may experience ambivalence, frustration, or loneliness related to the loss of their role. Caring for the patients, and with a high influx of new colleagues from different fields with limited or no previous experience of intensive care, led to frustration. Nurses in the current study expressed the frustration of not knowing each new individual rotated into the ICU, or the level of their competence. Knowing their new colleague had the responsibility to care for severely ill patients was both frustrating and caused stress. They identified a safety risk and knew that their new colleagues did not have the experience to value the severity of the patients’ health condition. The stress has been confirmed by nurses in other studies [9, 11, 13], as have the challenges of fragmented care, task-oriented groups, and skills-mix [14]. The nurses in the current study also knew that these professionals were put into managing situations without enough training. This is in line with research that describes challenges faced by the nurses in adapting to new team compositions and coping with an inflow of less experienced nurses. With a lack of a proper introduction, this became “learning by doing” [26]. In one study, different professions from three different sites suggested that new practitioners performing new tasks with indirect, or no supervision was common [12].

A transition can only appear if the individuals are aware of the ongoing changes. Following awareness, comes engagement, where the person immersed in the transition process undertakes activities such as seeking information or support to identify new ways of being, modifying former activities, and making sense of the circumstances [21]. In the transition phase, the participants try to adapt to a new situation [22]. The nurses in the current study gained experience by learning and reflecting on earlier activities and situations. They lived through the experience, and in time realized they had to shoulder the responsibility. They moved on from feelings of being put into a situation to adapting to the new situation and taking control of it, both in organizing and restructuring their work, as well as finding new collaborative structures with colleagues supporting each other at the ICU. They started to develop routines and to adapt to the new premises. It became evident that over time a sense of stability and new routines began to emerge, in line with Kralik [22], who suggests that transition is an ongoing process which involves movement in many directions. The transition process concludes with integration, which entails a new start and an inner reorientation [22]. In this stage, the understanding of new life circumstances is assimilated. The individual possesses the capacity to adapt to the altered situation [28]. As the last stage in the process, the integration phase occurred when the participants started to look forward and make long-term plans. It was a reorientation whereby they wanted to bring back the high-quality proactive and person-centred care, which to some extend had to be a lower priority during the pandemic. The nurses started to develop new structures and strategies to cope with the changed circumstances. Gratitude, cooperation and good teamwork were factors that were facilitated by the transition and also eased the process. Schumacher and Melies [20] describe circumstances that influence the way a person moves through a transition, and what factors, e.g., knowledge and attitudes, facilitate or hinder progress toward achieving a transition. The results from this study described that the participants had learned the importance of a secure and friendly work environment. They understood that they needed each other to get through together. They described that they become more sensitive and supportive of each other, offering encouragement, empathy, and understanding during these challenging times. A previous study described that working together during the pandemic created a way among the care team to form close relationships, characterized by strong interpersonal connections which brought the team together [27]. Indicators suggested for a healthy transition include feeling connected, interacting, and being situated [21]. The nurses in the current and other studies made efforts trying to help each other and other professions [9, 13]. To help each other was a given, and rarely reflected on. Studies indicate that healthcare teams frequently experience a sense of personal responsibility in addressing challenges [29, 30]. The teamwork was supportive, and participants had to rely on each other, but communication and IPC failed, most often between physicians and ICU nurses. This is in line with a previous study describing that collegial teamwork was crucial for ICU nurses, and that it was characterized by interdisciplinary collaboration, support and encouragement [13].

### Strengths and limitations

We ensured trustworthiness in this study by using several strategies and techniques. To improve credibility and confirmability, investigator triangulation was used [31]. All three authors independently analyzed each transcript and compared the findings to check for consistency. Differences were discussed and resolved until we reached full agreement. This process reduced the risk of bias and it ensured that the codes, themes, and subthemes were well-defined—externally diverse (distinct from each other) and internally consistent (cohesive within each theme) [24, 25]. Dependability was supported by the consistent data collection process, guided by a structured interview protocol and the analysis followed a well-defined research design [31]. To improve transferability, we provide a detailed description of the sampling methods, inclusion criteria, data collection, and analysis procedures. A reflective approach was adopted throughout the process where we critically examined our preconceptions and explicitly addressed potential biases. The team held multiple discussions to review and validate codes, to further reduce the risk of interpreter bias.

However, the study had some limitations. Online video conferencing was used for data collection due to the pandemic. While video communication was widely accepted at the time, it may have influenced participation. The study was conducted when ICU nurses were particularly overburdened and fatigued. If data collection had taken place after the pandemic slowed, more nurses might have participated. Despite this, the 16 nurses interviewed provided rich, in-depth descriptions, and the study achieved information power, indicating the data was sufficient for the analysis. Another limitation involves potential sampling bias. The nurses who volunteered for the study, despite their heavy workloads, may have had a particular interest in teamwork which could influence the findings.

## Conclusion

The findings capture the challenges ICU nurses went through during the pandemic, including losing the security of their ICU team, and having to adapt to constant changes to provide care to an increased number of critically ill patients. Initially, they felt alone and left to manage on their own without the support of their ICU team. However, they also experienced professional growth through adapting to contextual challenges and found new ways to collaborate. The challenges faced by nurses were significant and, they also demonstrated, adaptability, and a commitment to providing high-quality care. Their ability to adapt and being willing to find solutions helped nurses to regain a sense of control and manage the challenges they faced. Overall, the findings of this study provide insights into the transition process of the participating ICU nurses during the Covid-19 pandemic.

## Electronic supplementary material

Below is the link to the electronic supplementary material.

Supplementary Material 1

## Data Availability

Data can be shared upon reasonable request. Request of data (pseudoanonymized) can be sent to the Research Data Office (rdo@ki.se) at Karolinska Institutet, which will require a data transfer agreement and will be handled according to the relevant legislation in Sweden.

## Abbreviations

ICU: Intensive care unit
COREQ: Consolidated criteria for reporting qualitative research
