# Supplementary material for: Intensive care nurses’ experiences of teamwork during the covid-19 pandemic. a qualitative study
Source: BMC Nurs. 2025 Jan 27;24:97. doi: 10.1186/s12912-025-02696-8 (PMC11771016; doi:10.1186/s12912-025-02696-8)
Supplement: Supplementary file 1 — Supplementary Material 1 [file 12912_2025_2696_MOESM1_ESM.docx]

Appendix 1

Interview Questions

**How did intensive care change during the Covid-19 pandemic?**

*Follow-up questions*

-How did it function

-What was your role?

-Which professions were involved in the care?

-How did it feel about this?

- Do you remember any situations from this time?

-What were the challenges?

-How has this changed over the past years?

**How did the collaboration in your workgroup change during the Covid-19 pandemic?**

*Follow-up questions*

-Can you describe how the team functioned?

-What was your role?

-Who did you work with?

-Could you describe any situations from this time?

-How did you feel about this?

-Was there anything that could have facilitated your work?

-What were the challenges?

**How did your situation as an intensive care nurse change during the Covid-19 pandemic?**

*follow-up questions*

-Can you describe how you worked?

-Who did you work with?

-Could you describe any specific situations?

-How did you feel about this?

-Was there anything that could have facilitated your work?

-What were the challenges?
